# Rapid Progression of Skin Sclerosis Following Surgery for Carpal Tunnel Syndrome: A Case of Diffuse Cutaneous Systemic Sclerosis

**DOI:** 10.7759/cureus.47149

**Published:** 2023-10-16

**Authors:** Kota Sugisaki, Kiyonobu Sawamura, Mikako Ito, Keiko Kobayashi, Masao Hori

**Affiliations:** 1 Department of Rheumatology, Japanese Red Cross Mito Hospital, Mito, JPN; 2 Department of Dermatology, Japanese Red Cross Mito Hospital, Mito, JPN; 3 Department of Pathology, Japanese Red Cross Mito Hospital, Mito, JPN

**Keywords:** postoperative symptoms, complex regional pain syndrome, interstitial lung disease, carpal tunnel syndrome, diffuse cutaneous systemic sclerosis

## Abstract

Carpal tunnel syndrome (CTS) is a frequently encountered compressive neuropathy that is often treated surgically. Here, we present an unusual case of a 74-year-old female who developed a rapid emergence of skin sclerosis following CTS surgery. The condition was initially misdiagnosed as complex regional pain syndrome. However, since her skin condition progressed, she was referred to the rheumatology department. Subsequent evaluations confirmed the diagnosis of diffuse cutaneous systemic sclerosis, accompanied by interstitial lung disease. Treatment with mycophenolate mofetil did not notably alter the interstitial lung shadows but led to minor improvement in skin sclerosis. It is crucial to consider the possibility of rheumatic diseases in patients with unexpected postoperative symptoms.

## Introduction

Carpal tunnel syndrome (CTS) is a common compressive neuropathy of the median nerve, affecting a few percent to 10% of adults, frequently requiring surgical intervention for its treatment [1,2]. Although CTS is a commonly diagnosed condition, this report describes a distinctive case wherein a patient exhibited accelerated progression of skin sclerosis subsequent to CTS surgery. The patient was initially misdiagnosed with complex regional pain syndrome (CRPS), a disorder that often arises following minor trauma or surgical intervention and is characterized principally by the presence of persistent pain. This condition may additionally present with dermatological manifestations such as alterations in skin color changes and edema [3]. However, further examination revealed a conclusive diagnosis of diffuse cutaneous systemic sclerosis (dcSSc) accompanied by interstitial lung disease (ILD). This manuscript explores the potential triggers of such rapid dermatological alterations, including surgical trauma and the coronavirus disease 2019 (COVID-19) vaccination.

## Case presentation

A 74-year-old female presented to a local orthopedic clinic in March 2022 with complaints of numbness in both thumbs and fingers, extending to the ring fingers and palms. She was concerned about rheumatoid arthritis (RA) because her sister had also been diagnosed with the disease. No abnormalities were observed on plain radiographs of either hand. Although her C-reactive protein (CRP) level was slightly elevated (0.35 mg/dL (normal, less than 0.3)), she tested negative for rheumatoid factor and anti-cyclic citrullinated peptide antibodies, likely ruling out a diagnosis of RA. However, given her symptoms, CTS was suspected and she was referred to the orthopedic department of a nearby general hospital in June 2022. On evaluation, Tinel’s sign was positive bilaterally, as was Phalen’s test. A nerve conduction study (NCS) of the left median motor nerve revealed a slight prolongation of latency (4.28 ms), and nerve conduction velocity was at the lower limit of normal on both the right (49.0 m/s) and left sides (50.0 m/s). A mild reduction in conduction velocity was also observed in the median sensory nerves bilaterally, although with no prolongation of latency. Based on these physical findings and NCS results, the patient was diagnosed with bilateral CTS.

Following the diagnosis, conservative treatment with acetaminophen, pregabalin, and vitamin B12 was initiated but with no improvement in her symptoms. Therefore, surgical intervention was planned, and the patient underwent open carpal tunnel release surgery on her right hand in December 2022. Intraoperatively, the transverse carpal ligament was notably swollen, along with mild inflammatory hypertrophy of the synovium surrounding the median nerve. Pathological examination of the transverse carpal ligament was not performed. Postoperatively, the patient experienced a slight improvement in numbness, although it was not completely resolved.

In early January 2023, approximately two weeks after CTS surgery, the patient rapidly developed edematous skin sclerosis in both hands and forearms, accompanied by a reduction in the range of motion of her fingers. She was initially diagnosed with CRPS secondary to surgical intervention, and rehabilitation-focused treatment was initiated. However, her symptoms rapidly worsened and, by early February 2023, she experienced a significant decrease in grip strength, making it exceedingly difficult for her to use a towel or open a bottle cap. Since surgery had been performed only on her right hand, and the symptoms appeared bilaterally without any difference between the two sides, a condition other than CRPS was suspected. She was referred to the authors’ rheumatology department in mid-February, 2023.

**Figure 1:**
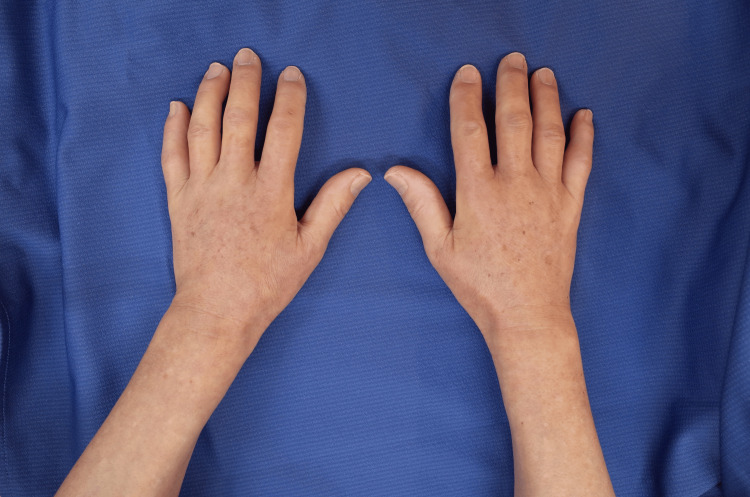
Patient's hands on the initial visit Manifestations of skin sclerosis and tautness extending from the fingers to the forearm, impeding skin pinchability and dramatically limiting the range of motion. There were no significant changes in the patient’s nails.

At her first visit to the department, edematous skin sclerosis was observed bilaterally from her fingers to her upper arms. Her fingers exhibited flexion contractures such that it was impossible for her to make a fist or bring her palms together completely (Figure 1).

**Figure 2:**
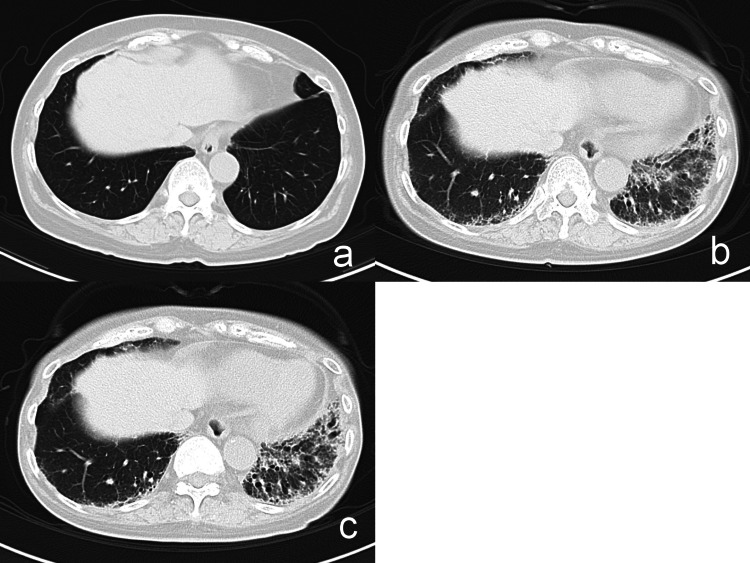
Chest computed tomography a: CT images in August 2017 reveal no noteworthy pulmonary lesions, including interstitial alterations. b: Before treatment, ground-glass opacities, reticular patterns, and traction bronchiectasis were discernible in both lower lung lobes, predominantly on the left side. c: After two months of treatment with mycophenolate mofetil, no considerable alterations were detected in the interstitial pattern in either lung.

No ulcers or pitted scars were observed on her fingers. Further questioning revealed that she had experienced Raynaud’s phenomenon for several years. No perioral wrinkles, tongue frenulum shortening, or nail fold bleeding were observed. Mild edematous skin sclerosis was also noted in both feet and legs. The modified Rodnan total skin thickness score was 30, with the highest score of 3 recorded for her fingers bilaterally, dorsal aspect of both hands, and both forearms. She had no fever, blood pressure was 142/86 mmHg, heart rate was 88 beats/min, and oxygen saturation (SpO2) was 96% on room air. The patient complained of pain in both knee joints although no inflammatory swelling was observed. No obvious rashes were noted, apart from skin sclerosis. She was able to eat independently, albeit with some difficulty, and had no gastroesophageal reflux or constipation. She had previously undergone left laparoscopic radical nephrectomy at the authors’ hospital for renal cell carcinoma at 50 years of age, after which she underwent periodic plain computed tomography (CT) of the chest and abdomen to ensure that there was no recurrence. The most recent imaging was performed in August 2017 and revealed no obvious local recurrence, metastasis, or interstitial lung lesions (Figure 2a).

She has been on amlodipine (5 mg/day) for hypertension since the age of 58 years. She had no history of diabetes mellitus. The patient had received three Pfizer/BioNTech COVID-19 vaccinations (COMIRNATY®) between May 2021 and August 2022 but experienced no side effects other than local pain. She had no history of COVID-19.

The initial laboratory findings are summarized in Table 1.

**Table 1:** Laboratory data on initial visit

	Results	Normal Range
White Blood Cell Count (/mm^3^)	5130	3000–8000
Hemoglobin (g/dL)	9.7	10–14
Platelets (× 10^4/mm^3^)	16.7	15–40
Blood Urea Nitrogen (mg/dL)	27.9	7–20
Creatinine (mg/dL)	1.25	0.4–0.8
C-Reactive Protein (CRP) (mg/dL)	0.13	< 0.3
Erythrocyte Sedimentation Rate (mm/h)	18	< 20
KL-6 (U/mL)	725	< 500
Antinuclear Antibody	1280 ×	< 40 ×
	(speckled and nucleolar patterns)	
Anti-SS-A	Negative	
Anti-SS-B	Negative	
Anti-DNA	Negative	
Anti-Sm	Negative	
Anti-RNP	Negative	
Anti-Scl-70	Negative	
Anti-RNA Polymerase 3 (index)	186	< 28

Mild anemia, a slight renal impairment likely attributed to left nephrectomy, and increased levels of KL-6 were observed. The antinuclear antibody (ANA) test was strongly positive, whereas anti-Scl-70 was negative. Notably, the anti-RNA polymerase 3 antibody, which is characteristic of dcSSc, was positive.

**Figure 3:**
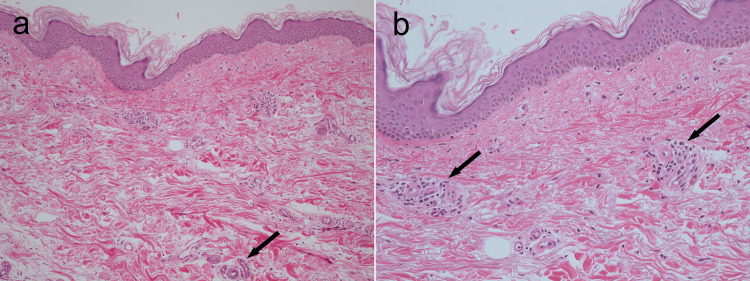
Histopathological examination of the patient’s right forearm (hematoxylin and eosin staining, original magnification ×50 (a) and ×100 (b)) a: The epidermis exhibited no particular changes, although the dermis exhibited eosinophilic fibrosis and atrophic sweat glands in deeper strata (arrows). b: Mild lymphocytic infiltration was present in the perivascular region (arrows).

Although the patient did not complain of coughing or shortness of breath, chest radiography revealed fine reticular shadows in both lower lung fields. Chest CT revealed ground-glass opacities, reticular shadows, and traction bronchiectasis in the lower lobes of both lungs, predominantly on the left side (Figure 2b). Spirometry did not reveal a significant decrease in percent vital capacity or forced expiratory volume in 1 s; however, the predicted diffusing capacity of the lungs for carbon monoxide (78.7%) was close to the lower limit of normal. Transthoracic echocardiography revealed no findings suggestive of pulmonary hypertension. Based on the above observations, dcSSc was suspected and a dermatologist was consulted. A skin biopsy of the right forearm was performed, which indicated pathological findings consistent with those of scleroderma (Figures 3a, 3b).

Based on these findings, the patient was diagnosed with dcSSc with ILD. Due to the subacute onset and presence of anti-RNAP3 antibodies, it was considered necessary to rule out paraneoplastic syndrome, and a whole-body search was performed; however, no malignancies were identified.

Prioritizing the treatment of ILD, which could have affected the patient’s activities of daily living and prognosis, administration of mycophenolate mofetil (MMF, 1000 mg/day) was initiated in late February 2023. In accordance with recent expert consensus guidelines on the management of systemic sclerosis-associated ILD, mycophenolate mofetil (MMF) was considered a suitable treatment option for this patient [4]. Additionally, given the patient's mild renal impairment attributed to left nephrectomy, MMF was favored over cyclophosphamide due to its comparatively better safety profile. While glucocorticoids are often considered for treating inflammatory conditions, they were intentionally avoided in this case. Given the known risk of inducing a scleroderma renal crisis, a life-threatening complication, especially when high doses are used, it was deemed prudent to opt for other treatment modalities. Chest CT in late April 2023 revealed no significant changes in the interstitial shadows (Figure 2c); however, a slight improvement was observed in skin sclerosis on her fingers. Due to the limited improvement in ILD despite MMF treatment, nintedanib was added to the therapeutic regimen.

## Discussion

CTS is a compressive neuropathy of the median nerve in the carpal tunnel resulting from various etiologies. It is considered to be the most common mononeuropathy, with numerous reports estimating its prevalence as ranging from a few percent up to 10% [1,2]. Although the majority of cases are idiopathic, known causes include synovitis from RA or amyloid deposition in patients undergoing hemodialysis. In fact, the odds ratio for CTS onset in patients with RA is 2.2 [5]. Additionally, CTS is highly prevalent in patients with diabetic neuropathy and nerve vulnerability [6], pregnant women [7], and postmenopausal women [8]. The recommended method for diagnosing CTS is NCS [9], as was used for making a definitive diagnosis in this case. The treatment typically involves pharmacotherapy for mild cases and surgery for more severe cases, although approximately one-third of patients reportedly experience spontaneous improvement [10].

There have been a few reported cases in which CTS was recognized as one of the symptoms of systemic sclerosis [11-13]. However, to the best of our knowledge, our report is the first to describe pathologically confirmed skin sclerosis that rapidly manifested after CTS surgery. Very few reports have described cases in which rheumatic diseases, besides CTS and systemic sclerosis, have presented anew after surgery [14-16]. Among these, thymectomy in patients with myasthenia gravis (MG) is known to increase the risk of developing rheumatic diseases [17]. However, this is likely due to immunological mechanisms caused by the loss of thymic function rather than surgical trauma itself being a trigger. In contrast, while the exact mechanism in our case remains elusive, we cannot rule out the possibility that the surgical intervention for CTS might have played a role as a potential trigger for the rapid progression of skin sclerosis.

In this patient, ILD did not exhibit a clear response to two months of treatment with MMF. It is possible that the ILD had already passed the stage at which it could respond to immunosuppressive therapy, making it difficult to determine whether it emerged simultaneously with skin sclerosis. There have been a few reported cases of systemic sclerosis in which ILD preceded skin sclerosis, and it is possible that our case falls into this category [18,19]. We speculate that the ILD existed independently of the CTS surgery and had been present for some time. In this case, Raynaud’s phenomenon was the only symptom definitely present before CTS surgery, suggesting its correlation with dcSSc.

Our patient received three doses of the COVID-19 vaccine before the onset of dcSSc. We believe it is important to consider a potential correlation between vaccination and the onset of dcSSc. This is because recently, various types of autoimmune diseases, such as MG [20,21], systemic lupus erythematosus [22-24], dermatomyositis [25], psoriasis vulgaris [26], immunoglobulin A vasculitis [27,28], cutaneous leukocytoclastic vasculitis [29-31], antineutrophil cytoplasmic antibody-associated vasculitis [32,33], and polyarteritis nodosa [34], have been reported to develop anew after COVID-19 vaccination. We were unable to find any large-scale studies that quantitatively assess the incidence of new-onset rheumatic diseases post-vaccination. However, it is worth noting that rheumatic diseases triggered by COVID-19 vaccination have been reported to generally respond well to standard treatments and have a relatively favorable prognosis [35]. Within the scope of our search, we found only one case report of new-onset dcSSc following COVID-19 vaccination [36]. That report described a case involving a 70-year-old male who developed rapidly progressive skin sclerosis two weeks after the first vaccination. Similar to our case, the patient was strongly positive for ANA, although no disease-specific autoantibodies were detected. Akkuzu et al. reviewed reported cases of newly developed rheumatic diseases after COVID-19 vaccination and found that the onset of new rheumatic diseases ranged from four to 90 days after the final vaccination, with more than half the cases occurring after the first vaccination [37]. In our case, about five months elapsed between the last vaccination and the onset of dcSSc, which was a longer duration than in previously reported cases. Therefore, we speculated that surgical invasion likely contributed more strongly to dcSSc than COVID-19 vaccination.

In this case, the patient was initially diagnosed with CRPS due to the rapid emergence of skin sclerosis following CTS surgery, although aggressive rehabilitation proved ineffective. CRPS is a syndrome in which there is disproportionate pain persistence compared to the degree of tissue damage after trauma or surgery. It is characterized by abnormalities in the skin, including changes in skin color, sclerotic edema, contractures, abnormal skin temperature, and sweat disorders [3]. Typically, CRPS manifests unilaterally, centering on the site of injury. While there are instances of bilateral presentation, such as the case reported by Kong ST et al. where CRPS developed bilaterally after cervical spinal cord injury [38], these are deemed atypical and occur under specific circumstances. In our patient, the onset and progression of skin sclerosis were symmetrical on both sides, distinct from the usual clinical presentation of CRPS. Given the unique features of our patient's case, such as the bilateral and symmetrical onset of skin sclerosis, the initial diagnosis of CRPS may have been incorrect, although all types of trauma, including hand surgery, have the potential to precipitate CRPS [39].

## Conclusions

We encountered an extremely rare case of the rapid manifestation of skin sclerosis after CTS surgery. The possibility of rheumatic diseases should be considered in cases with unexpected postoperative symptoms.
